# Sildenafil ameliorates right ventricular early molecular derangement during left ventricular pressure overload

**DOI:** 10.1371/journal.pone.0195528

**Published:** 2018-04-05

**Authors:** Yousuke Imai, Taro Kariya, Masaki Iwakiri, Yoshitsugu Yamada, Eiki Takimoto

**Affiliations:** 1 Department of Anesthesiology, Graduate School of Medicine, The University of Tokyo, Bunkyo-ku, Tokyo, Japan; 2 Department of Cardiovascular Medicine, Graduate School of Medicine, The University of Tokyo, Bunkyo-ku, Tokyo, Japan; 3 Division of Cardiology, Department of Medicine, Johns Hopkins University School of Medicine, Baltimore, Maryland, United States of America; Scuola Superiore Sant'Anna, ITALY

## Abstract

Right ventricular (RV) dysfunction following left ventricular (LV) failure is associated with poor prognosis. RV remodeling is thought initiated by the increase in the afterload of RV due to secondary pulmonary hypertension (PH) to impaired LV function; however, RV molecular changes might occur in earlier stages of the disease. cGMP (cyclic guanosine monophosphate)-phosphodiesterase 5 (PDE5) inhibitors, widely used to treat PH through their pulmonary vasorelaxation properties, have shown direct cardiac benefits, but their impacts on the RV in LV diseases are not fully determined. Here we show that RV molecular alterations occur early in the absence of RV hemodynamic changes during LV pressure-overload and are ameliorated by PDE5 inhibition. Two-day moderate LV pressure-overload (transverse aortic constriction) neither altered RV pressure/ function nor RV weight in mice, while it induced only mild LV hypertrophy. Importantly, pathological molecular features were already induced in the RV free wall myocardium, including up-regulation of gene markers for hypertrophy and inflammation, and activation of extracellular signal-regulated kinase (ERK) and calcineurin. Concomitant PDE5 inhibition (sildenafil) prevented induction of such pathological genes and activation of ERK and calcineurin in the RV as well as in the LV. Importantly, dexamethasone also prevented these RV molecular changes, similarly to sildenafil treatment. These results suggest the contributory role of inflammation to the early pathological interventricular interaction between RV and LV. The current study provides the first evidence for the novel early molecular cross-talk between RV and LV, preceding RV hemodynamic changes in LV disease, and supports the therapeutic strategy of enhancing cGMP signaling pathway to treat heart diseases.

## Introduction

The right ventricle (RV) is a chamber of the heart that pumps out blood into pulmonary circulation under low pressure. While cardiac output can be maintained even in the absence of functional RV under physiological conditions, growing evidence has indicated that RV dysfunction has deleterious impacts on prognosis as well as functional capacity in heart failure [1,2]. In patients with systolic heart failure, RV ejection fraction (RVEF) <20% is an independent predictor of mortality and heart failure hospitalization [3]. RV dysfunction is the strong predictor of death also in heart failure patients with preserved ejection fraction (HFpEF), associated with severe symptoms [4,5]. Although RV dysfunction following LV disease is thought initiated due to RV pressure-overload, which occurs following elevated LV end-diastolic pressure, with the mechanisms shared by pulmonary arterial hypertension or pulmonary stenosis, studies that directly assess molecular mechanisms remain scant.

Cyclic guanosine monophosphate (cGMP) is an intracellular second messenger downstream of nitric oxide and natriuretic peptides, and has been gaining attention as a key to heart failure treatment. Phosphodiesterase 5 (PDE5) inhibitors block degradation of cGMP and thus activate cGMP signaling pathways. While PDE5 inhibitors are in wide clinical use for the treatment of pulmonary hypertension, benign prostate hyperplasia and erectile dysfunction [6] through their vasorelaxation action, growing evidence has suggested that PDE5 inhibition also provide beneficial cardiac effects. PDE5 inhibition with sildenafil or tadalafil ameliorated experimental models of heart diseases in rodents [7,8]. Chronic sildenafil treatment improved cardiac function and clinical status in patients with systolic heart failure and diabetic cardiomyopathy [9,10]. Prior studies revealed that multiple mechanisms might contribute to such cardiac benefits, including Gq signal deactivation [8], improvement of mitochondrial energy metabolism [11] and modulation of inflammation [12]; however, the molecular impact of PDE5 inhibition on RV has not been fully determined.

In the current study, employing a mouse model of LV pressure-overload, we investigated early molecular changes in the RV myocardium, and tested the impacts of concomitant sildenafil treatment. We found that pathologic molecular derangement in the RV myocardium occurred at very early stages before RV hemodynamic overload became evident, and that sildenafil ameliorated such molecular abnormalities though mechanisms involving its anti-inflammatory effects.

## Material and methods

### Ethics statement

All animal protocols were approved by the animal care and use committee of the University of Tokyo (approval number: H15-099).

### Animal models

All experiments were performed on C57BL/6J male mice (7–10 weeks old; CLEA Japan, Tokyo, Japan). They were housed in controlled environment with a 12h light/ 12h dark cycle at a maintained temperature, and kept with free access to food and water throughout the whole experiment period. Pressure overload was posed by transverse aortic constriction (TAC) [7]. We prepared four arms: (1) sham surgery (Sham), (2) TAC with normal soft chow (TAC 2d Veh), (3) TAC with sildenafil chow (TAC 2d Sil), and (4) TAC with dexamethasone treatment (TAC 2d DXM). Animals were anesthetized with 1% inhaled isoflurane and 10 mg/kg intraperitoneal etomidate, then intubated, and mechanically ventilated. The mediastinum was opened through dislocation of 2nd and 3rd left sternocostal joints, then transverse aorta was exposed at the back of thymus. Between the brachiocephalic trunk and the left common carotid artery a 27-gauge needle was placed alongside transverse aorta, and the aorta and the needle was tied around using 7–0 prolene suture. After the needle was withdrawn, the aorta was constricted to a diameter of 0.4 mm. Sham-operated animals were subjected to the same surgical procedures without aortic constriction. After the chest closure with 6–0 prolene, they were allowed to recover from anesthesia, and placed on a heating plate until full recovery of consciousness. For two days after surgery TAC-2d-Sil mice were treated with sildenafil citrate (Wako Pure Chemical Industries, Osaka, Japan; 200mg/kg/day) mixed in soft chow (Transgenic Dough Diet; Bio-Serv, Flemington, NJ; 100g/kg/day). Free plasma concentration of sildenafil with the dose in mice are comparable to those in humans using standard clinical dosing[7], because mice metabolize sildenafil at approximately 100 times higher rate than humans[13]. Sham, TAC-2d-Veh and TAC-2d-DXM animals received the soft diet without sildenafil. TAC-2d-DXM mice were injected intraperitoneally with dexamethasone sodium phosphate (Aspen Japan, Tokyo, Japan; 20mg/kg/day) on the day of the surgery and the following day. Total number of mice used in this study was 38. In each group (Sham, TAC 2d Veh, TAC 2d Sil), eleven mice were allocated. Out of 11, three mice for in vivo hemodynamics study, five for gene expression study, and three for western blot analysis. Five mice were allocated to TAC-2d-DXM group for gene expression study.

### Echocardiography

Before and two days after surgery cardiac function was assessed by transthoracic, two-dimensional guided M-mode echocardiography in conscious mice using Vevo2100 (FUJIFILM VisualSonics, Toronto, Ontario, Canada) with 30 MHz linear-array transducer. M-mode LV end-systolic diameter (LVESD) and LV end-diastolic diameter (LVEDD) were measured in the short-axis view. LV fractional shortening (LVFS) was calculated as follows: LVFS = (LVEDD-LVESD)/LVEDD. Studies and analysis were performed by the same investigator (TK).

### *In vivo* hemodynamics

For more comprehensive analysis, *in vivo* LV and RV function were assessed by pressure catheter. Mice were anesthetized with 0.5% inhaled isoflurane, 1000 mg/kg intraperitoneal urethane, and 10 mg/kg intraperitoneal etomidate, were subjected to tracheostomy, and were ventilated with 6–7 μl/g tidal volume and 120 breaths/min. Volume loading (10% bovine albumin, 100–150 μl over 3 minutes) was provided via a 30-gauge cannula in the left external jugular vein. The LV apex was exposed by incising diaphragm and left costal arch. A 1.4-Fr pressure catheter (SPR-839; Millar Instruments, Houston, TX) was inserted through the LV apex which was in advance pricked with a 26-gauge needle, and was positioned along the longitudinal axis. And a 1-Fr pressure catheter (PVR-1035; Millar Instruments) was placed into the RV which was in advance pricked with a 27-gauge needle. All data were collected, saved to disk, and analyzed using MPVS Ultra Foundation System (ADInstruments, New South Wales, Australia) with LabChart 7 software (ADInstruments). All values were averaged over nine consecutive cardiac cycles while blood pressure was stable.

### Tissue preparation

After physiological studies, mice were euthanized by cervical dislocation, and the heart was resected and washed in phosphate buffered saline (PBS; 137.0mM NaCl, 2.68mM KCl, 8.1mM Na_2_HPO_4_, and 1.47mM KH_2_PO_4_, pH 7.4). Total heart tissues for immunohistochemistry were proceeded to fixation steps, and those for gene expression analysis and western blotting were dissected as below. After total heart weight was measured, great vessels and atriums were removed, the RV free wall was separated from the LV wall and the intraventricular septum (IVS), and RV free wall was weighed. LV apex and IVS were removed from the LV. Remained LV free wall and the RV free wall were trimmed 0.5 mm from edges. Each part of a heart was rapidly frozen in liquid nitrogen, then stored at -80°C for subsequent procedures. The lung weight and tibial length was measured.

### Quantitative real-time polymerase chain reaction (PCR)

Total ribonucleic acid (RNA) was extracted from frozen heart tissue using TRI Reagent (Molecular Research Center, Cincinnati, OH) according to the manufacturer's instructions. The yield of RNA was estimated spectrophotometrically using Nanodrop 2000 (Thermo Fisher Scientific, Waltham, MA). The same amount of RNA was reverse transcribed into complementary deoxyribonucleic acid (cDNA) using High Capacity RNA-to-cDNA kit (Thermo Fisher Scientific).

Quantitative real-time PCR was carried out using SYBR Green assay or Taqman probe assay. SYBR Green assay was conducted using THUNDERBIRD SYBR qPCR Mix (TOYOBO, Osaka, Japan) according to the manufacturer's instructions. The following primers were used: mouse Bnp 5’-AAGTCCTAGCCAGTCTCCAGA-3’ (forward) and 5’-GAGCTGTCTCTGGGCCATTTC-3’ (reverse); mouse B-MHC 5’-ATGTGCCGGACCTTGGAAG-3’ (forward) and 5’-CCTCGGGTTAGCTGAGAGATCA-3’ (reverse); and mouse Gapdh 5’-CATGGCCTTCCGTGTTCCTA-3’ (forward) and 5’-CCTGCTTCACCACCTTCTTGAT-3’ (reverse). PCR conditions were 2 minutes at 50°C, 2 minutes at 95°C, and 40 cycles of 95°C for 15 seconds and 60°C for 1 minute. Taqman assay was performed using THUNDERBIRD Probe qPCR Mix (TOYOBO) with following TaqMan primers (Thermo Fisher Scientific): mouse Il1b (Assay ID: Mm00434228_m1), mouse Il6 (Assay ID: Mm00446190_m1), mouse Nox2 (Assay ID: Mm01287743_m1), mouse Nox4 (Assay ID: Mm00479246_m1), mouse Rcan1 (Assay ID: Mm01213407_m1), and mouse Gapdh (Assay ID: Mm99999915_g1). Thermal cycling was 2 minutes at 50°C, 10 minutes at 95°C, and 40 cycles of 95°C for 15 seconds and 60°C for 1 minute. PCR reactions were performed, recorded, and analyzed by using LightCycler 480 (Roche Applied Science, Mannheim, Germany) or QuantStudio 5 (Thermo Fisher Scientific). PCR was performed in duplicate and gene expression level was normalized by Gapdh.

### Western blot analysis

Briefly, frozen heart tissues were homogenized in Cell Lysis Buffer (Cell Signaling Technology, Danvers, MA) with 1 mM phenylmethylsulfonyl fluoride, proteinase inhibitors cocktail (cOmplete Mini EDTA-free; Roche Applied Science), and phosphatase inhibitors cocktail (PhosSTOP; Roche Applied Science). The homogenate was centrifuged at 17,800 *g* for 10 minutes at 4°C, and the resulting supernatant was designated proteins fraction. Protein concentration of each fraction was measured using BCA Protein Assay Reagent (Thermo Fisher Scientific), and samples were diluted to the same concentration. Each protein solution was added with NuPage LDS Sample Buffer (Thermo Fisher Scientific) and 0.1M dithiothreitol, denatured at 95°C for 10 minutes, then sample solutions were obtained. Sample solutions were separated on 12.5% polyacrylamide gels (Wako Pure Chemicals Industries), and transferred onto the polyvinylidene difluoride membrane (Bio-Rad, Hercules, CA). Blotted membranes were blocked for 1 hour at room temperature with 5% nonfat dried milk in Tris-buffered saline solution (TBS; 20mM Tris, 500mM NaCl, pH 7.4) containing 0.1% Tween-20 (Sigma-Aldrich, St. Louis, MO). Blocked membranes were incubated overnight at 4°C with following primary antibodies: 1:1,000 of anti-Erk1/2 (#9102; Cell Signaling Technology), and 1:1,000 of anti-phospho-Erk1/2 (#9101; Cell Signaling Technology). Next day, the membranes were incubated with horseradish peroxidase conjugated secondary antibody (1:5,000) (sc-2357; Santa Cruz Biotechnology, Dallas, TX) for 1 hour at room temperature. Immunoreactive bands were developed using enhanced chemiluminescence substrate (SuperSignal West Femto Maximum Sensitivity Substrate; Thermo Fisher Scientific) and visualized by LAS-4000 mini imaging system (Fujifilm, Tokyo, Japan). Immunoblot bands was quantified using ImageJ software (NIH Image, Bethesda, MD).

### Immunohistochemistry

Total heart tissues were fixed in Tissue-Tek UFIX (Sakura Finetek Japan, Tokyo, Japan), embedded in paraffin, and cut cross-sectionally into 4–6 μm slices. Tissue sections were deparaffinized with a series of xylene washes, and rehydrated in ethanol solutions with decreasing concentrations from 100% to 90%, 80%, and 70%. Antigen retrieval was carried out by incubating the sections with 0.1% trypsin solution (Sigma-Aldrich) for 40 minutes at 37°C. Endogenous peroxidases were quenched in 3% of hydrogen peroxide in methanol for 10 minutes at room temperature. Subsequently, slides were blocked for 1 hour at room temperature with PBS containing 1.5% of normal rabbit serum (S-5000, Vector Laboratories, Burlingame, CA), and incubated overnight at 4°C with 1:400 of rat anti-mouse F4/80 antibody (MCAP 497; Bio-Rad, formerly AbD Serotec). The following day, rabbit anti-rat biotinylated secondary antibody (BA-4000, Vector Laboratories) was diluted 1.5:100 in 0.5% normal rabbit serum, and the sections were incubated for 1 hour at room temperature. Then slides were treated with avidin-peroxidase conjugate (VECTASTAIN Elite ABC Kit; PK-6104; Vector Laboratories) for 30 minutes at room temperature, and the signal was developed using the 3,3’-diaminibenzidine (DAB) substrate (SK-4100; Vector Laboratories). After counterstaining with hematoxylin, slides were dehydrated through graded ethanol (95% and 100%), cleared by washing in xylene, and mounted with Mount Quick (Daido Sangyo, Tokyo, Japan). Slides were examined by light microscopy (BX51; Olympus, Tokyo, Japan) and the number of F4/80 positive cells was counted in 10 randomly selected high-power field (400x magnification) with the observer (TK) blinded to sample identity. All the images were obtained at 200x magnification using an Olympus DP70 camera.

### Statistical analysis

All values are expressed as mean ± standard error of the mean (s.e.m). Differences between multiple groups were compared by one-way or two-way analysis of variance (ANOVA) and Tukey’s post-hoc multiple-comparisons test, using EZR (Saitama Medical Center, Jichi Medical University, Saitama, Japan) which is a graphical user interface for R (The R Foundation for Statistical Computing, Vienna, Austria, version 3.3.1) [14]. The other analyses were performed with Excel 2013 (Microsoft, Redmond, WA). P values less than 0.05 were considered significant.

## Results

### RV remained apparently normal by two-day LV pressure overload (TAC), with mild LV hypertrophy that was inhibited by sildenafil

**Fig 1:**
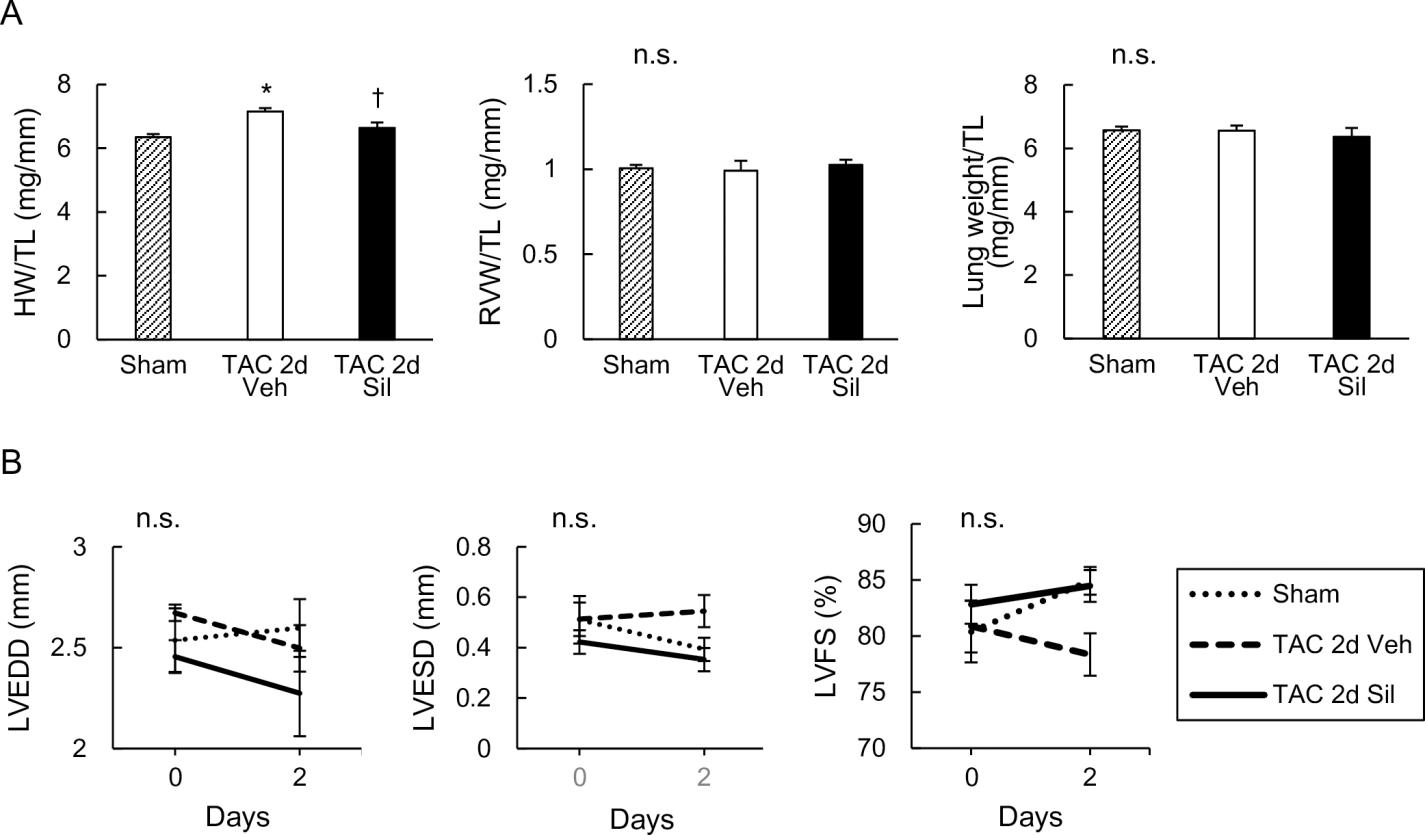
Transverse aortic constriction for two days increased heart weight, but did not affect echocardiographic parameters. (A) Postmortal assessment of heart, ventricles, and lung. Heart weight (HW), RV weight (RVW), and lung weight normalized to tibial length (TL) is shown. Transverse aortic constriction (TAC) for two days increased HW, and sildenafil prevented this increase. Neither RVW nor lung weight was affected by two-day TAC with or without sildenafil treatment. (B) Time course of echocardiographic parameters. LV end-diastolic dimension (LVEDD), LV end-systolic dimension (LVESD), and LV fractional shortening (LVFS) are shown. Two-day TAC altered neither LV dimensions nor function. Results are expressed as mean ± s.e.m. (n = 5). TAC 2d Veh, TAC for 2 days with vehicle treatment; TAC 2d Sil, TAC for 2days with sildenafil treatment. n.s., not significant by one-way or two-way analysis of variance; *, p<0.05 versus sham group; †, p<0.05 versus TAC 2d Veh group.

To examine early molecular alterations in the RV following LV disease, we used a mouse model of LV pressure-overload (TAC), in which LV was exposed to moderate pressure-overload created by surgical constriction of transverse aorta and examined the effect of concomitant sildenafil treatment. Two-day TAC induced only mild increase in the total heart weight (~10%), and this was inhibited by sildenafil treatment (Fig 1A). At this early stage, RV free wall weight and lung weight was neither impacted by TAC nor by sildenafil treatment (Fig 1A). M-mode transthoracic echocardiography showed minimal change in LV chamber size (LVEDD: LV end-diastolic dimension; LVESD: LV end-systolic dimension) and function (LVFS: LV fractional shortening) (Fig 1B). These results indicate that RV was apparently unaffected at this early stage when LV developed hypertrophy in response to the loading stress.

### Two-day LV pressure overload (TAC) did not affect RV pressure or function, but prolonged LV relaxation that was ameliorated by sildenafil

**Fig 2:**
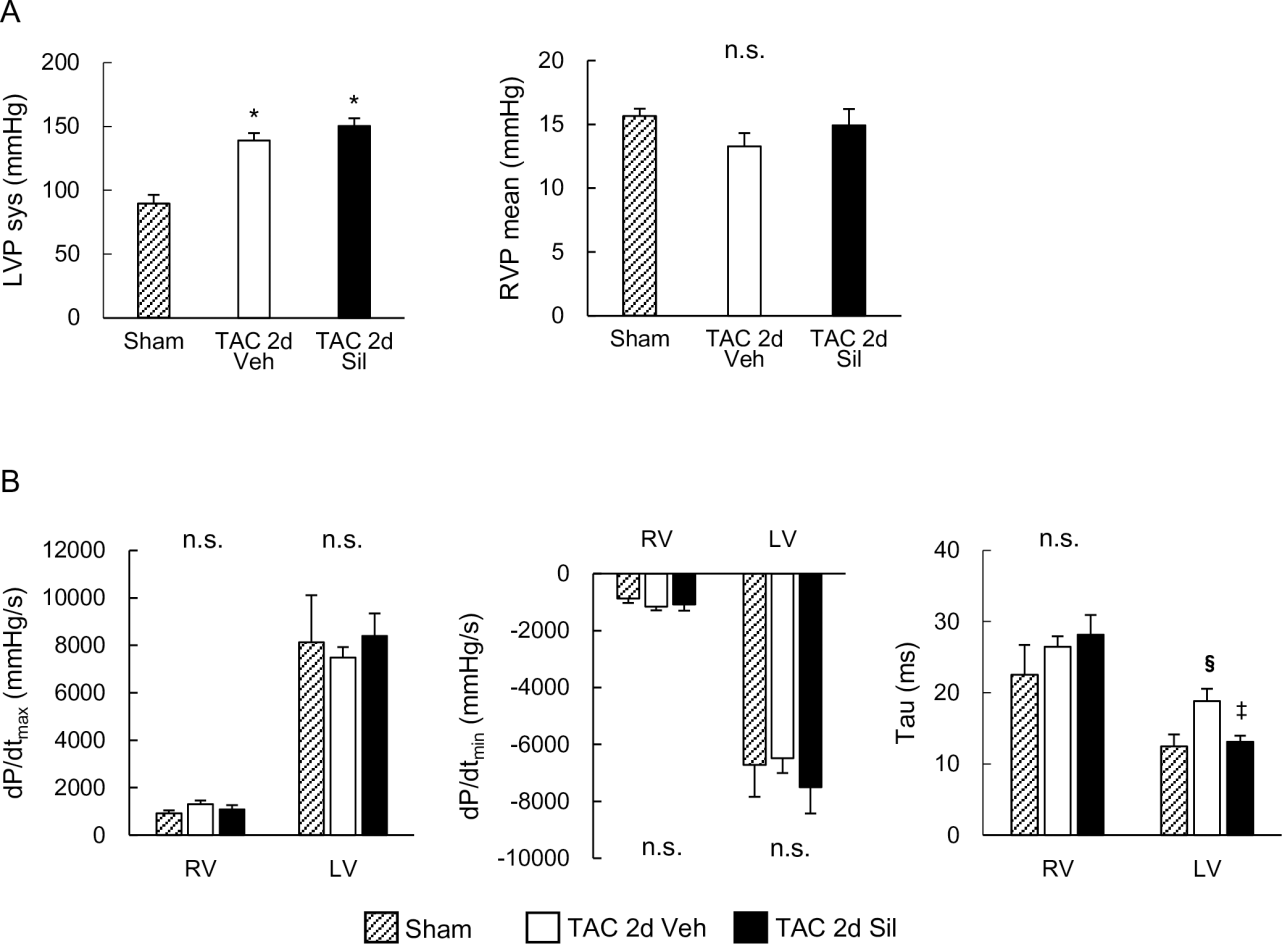
Invasive hemodynamic studies revealed two-day transverse aortic constriction affect neither right ventricular pressure nor function. (A) Peak systolic left ventricular pressure (LVP sys) and mean right ventricular pressure (RVP mean). Transverse aortic constriction (TAC) for two days increased systolic LV pressure, but had no effects on RV pressure. Sildenafil did not affect either pressure. (B) Peak rate of ventricular pressure rise (dP/dt_max_), peak rate of ventricular decline (dP/dt_min_), and relaxation time constant (tau). LV tau was prolonged by two-day TAC, which was normalized by sildenafil. Results are expressed as mean ± s.e.m. (n = 3). TAC 2d Veh, TAC for 2 days with vehicle treatment; TAC 2d Sil, TAC for 2days with sildenafil treatment. n.s., not significant by one-way analysis of variance; *, p < 0.05 versus sham group; §, p = 0.05 versus sham group; ‡, p = 0.07 versus TAC 2d Veh group.

We also performed hemodynamic studies using a micro-catheter placed in both ventricles for detailed hemodynamic assessment. While TAC induced significant increase in LV peak systolic pressure (LVP sys, 139mmHg), compared to sham controls (89 mmHg), RV mean pressure (RVP mean) was not yet affected (Fig 2A). These results confirm the absence of RV hemodynamic load at this early stage of the LV disease caused by moderate LV pressure-overload. Concomitant sildenafil treatment did not lower systolic LV peak pressure as reported previously [15], or affect RV mean pressure (Fig 2A). Neither dP/dt_max_ (peak rate of ventricular pressure rise) nor dP/dt_min_ (peak rate of ventricular pressure decline) was altered by the moderate TAC in either ventricles at this early stage (Fig 2B). Importantly, however, relaxation time constant (tau) was prolonged in the LV, again consistent with the general notion that the impairment of LV relaxation occurs early before systolic function starts to deteriorate in heart diseases. Sildenafil treatment normalized LV tau, while the other parameters examined were not altered (Fig 2B).

### RV revealed early hypertrophic molecular changes similar to LV during LV pressure-overload, and sildenafil inhibited these in both ventricles

**Fig 3:**
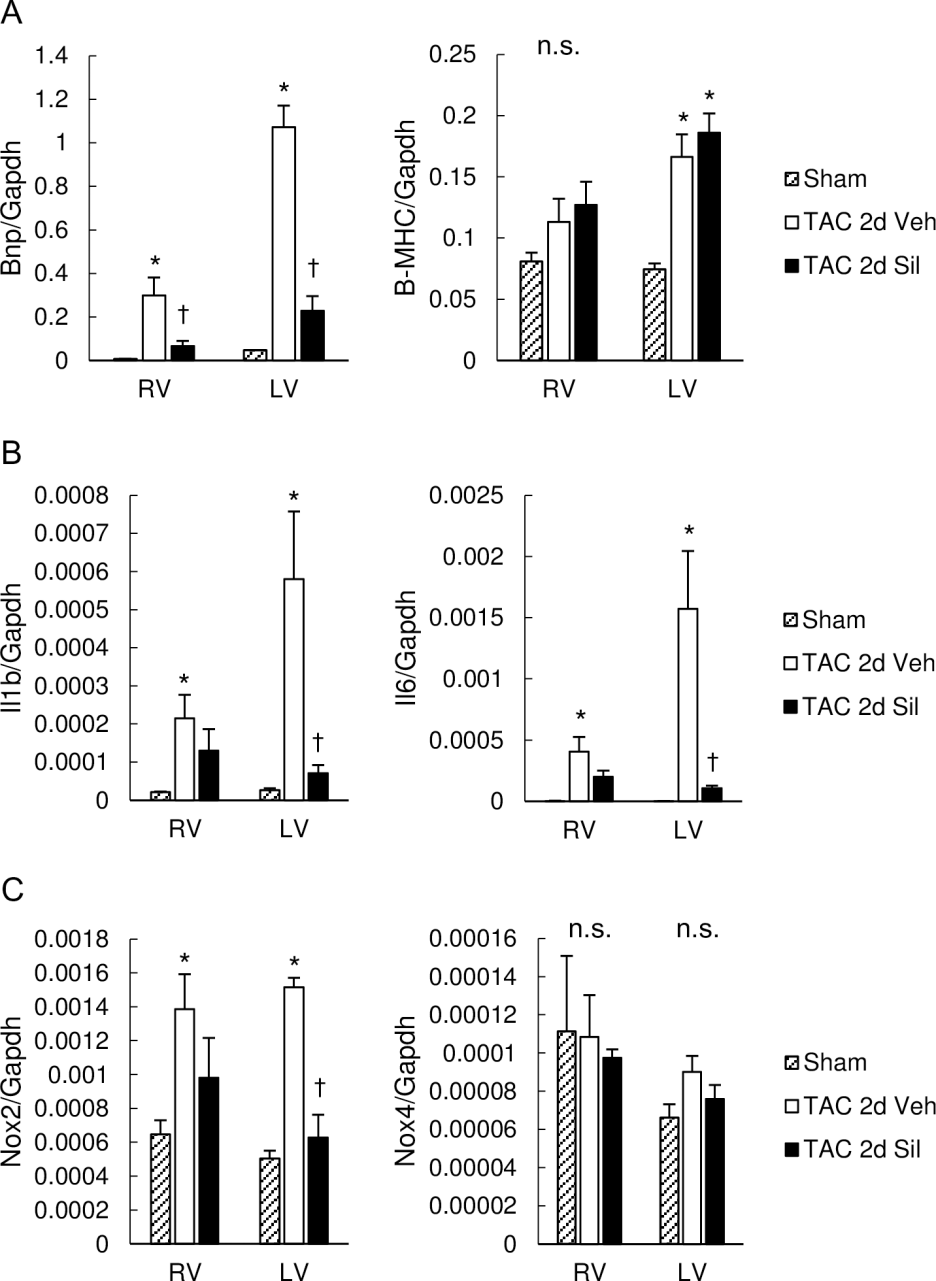
Pathological gene induction in RV as well as LV were ameliorated by sildenafil. (A) Expression of fetal genes as markers of cardiac hypertrophy, encoding for BNP and B-MHC, normalized to GAPDH. Transverse aortic constriction (TAC) for two days induced marked increase in BNP mRNA levels in the RV myocardium as well as in the LV myocardium, which was prevented by sildenafil. Expression of B-MHC was increased by two-day TAC in LV, but was not affected by sildenafil. (B) Expression of inflammatory cytokines, IL1b and IL6, normalized to GAPDH. IL1b and IL6 was up-regulated by TAC in both ventricles. Sildenafil inhibited both in LV, and also attenuated both in RV. (C) Expression of genes for enzymes inducing oxidative stress, NOX2 and NOX4, normalized to GAPDH. Expression of NOX2 was increased by TAC in both ventricles, and suppressed by sildenafil in LV. TAC and sildenafil had little influence on NOX4 expression. Results are expressed as mean ± s.e.m. (n = 5). TAC 2d Veh, TAC for 2 days with vehicle treatment; TAC 2d Sil, TAC for 2days with sildenafil treatment. n.s., not significant by one-way analysis of variance; *, p<0.05 versus sham group; ✝, p<0.05 versus the TAC 2d Veh group.

To elucidate molecular changes in both ventricles, we first determined messenger RNA (mRNA) expression levels for brain natriuretic peptide (BNP) and beta myosin heavy chain (B-MHC), both markers for fetal gene recapitulation. Interestingly, BNP mRNA levels were markedly increased in the RV free wall myocardium as well as in the LV myocardium in two-day TAC hearts (Fig 3A). Sildenafil treatment potently inhibited BNP expression levels in both ventricles, whereas it had no impact on the latter. Interestingly, pro-inflammatory marker genes, including interleukin-1 beta (IL1b) and interleukin-6 (IL6), were also up-regulated in the RV free wall as well as in the LV myocardium (Fig 3B), associated with increased nicotinamide adenine dinucleotide phosphate oxidase 2 (NOX2) but not NOX4 mRNA levels (Fig 3C), suggesting the involvement of inflammation as well as oxidative stress in the RV and LV pathophysiology at this early stage of the LV disease. Sildenafil treatment significantly inhibited the mRNA increase in IL1b, IL6 and NOX2 in LV, and also attenuated the increase in these genes in RV (Fig 3B and 3C). These results suggest the anti-inflammatory and anti-oxidative impacts of sildenafil.

### ERK and calcineurin were activated in the RV as well as in the LV, and were inhibited by sildenafil treatment

**Fig 4:**
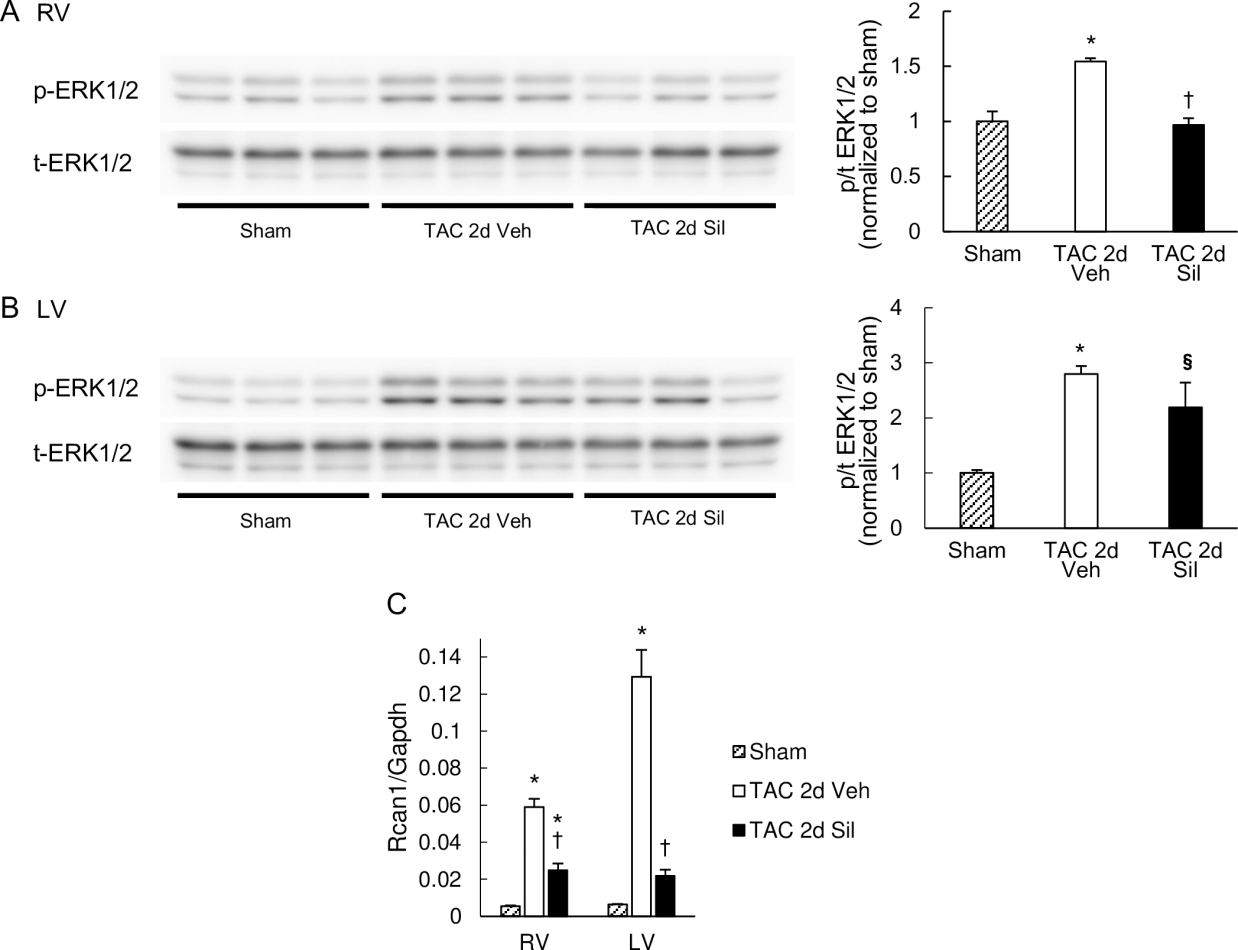
Hypertrophic signaling pathways were activated also in the RV, which was suppressed by sildenafil. Western blots for phosphor- (p-) and total- (t-) ERK1/2 in the RV (A) and the LV (B). Quantification results of phosphor/total ratio (p/t ratio) normalized to sham controls are shown in the bar graphs on the right. TAC induced robust phosphorylation of ERK1/2 in RV as well as LV myocardium, and sildenafil prevented this activation in the RV. Results are expressed as mean ± s.e.m. (n = 3). (C) RCAN1 expression normalized to GAPDH. RCAN1 mRNA expression was increased by TAC in both ventricles, and suppressed by sildenafil. Results are expressed as mean ± s.e.m. (n = 5). TAC 2d Veh, TAC for 2 days with vehicle treatment; TAC 2d Sil, TAC for 2days with sildenafil treatment. *, p<0.05 versus sham group; §, p = 0.050 versus sham group; ✝, p<0.05 versus the TAC 2d Veh group.

We also examined RV hypertrophic signaling pathways at this early stage of LV disease, including ERK and calcineurin; the former known to contribute to concentric hypertrophy and the latter to pathologic remodeling [16–18]. For calcineurin activity, we assessed RCAN1 (regulator of calcineurin 1) gene expression levels. RCAN1 is extremely responsive to changes in calcineurin activity in vivo [19] and thus its expression levels have been used as reflecting calcineurin activity [20,21]. ERK was significantly phosphorylated in the RV as well as in the LV (Fig 4A and 4B), and calcineurin activity was also increased in the RV similarly to the LV (Fig 4C). Sildenafil treatment significantly inhibited both these signals in the RV and the LV (Fig 4). These results suggest that the RV undergoes early hypertrophy molecular changes similar to the LV in the absence of RV afterload increase at the very early stage of LV pressure-overload, and that sildenafil treatment inhibits such molecular remodeling process in both ventricles.

### Sildenafil prevented cardiac macrophage infiltration in the RV as well as in the LV during LV pressure-overload

**Fig 5:**
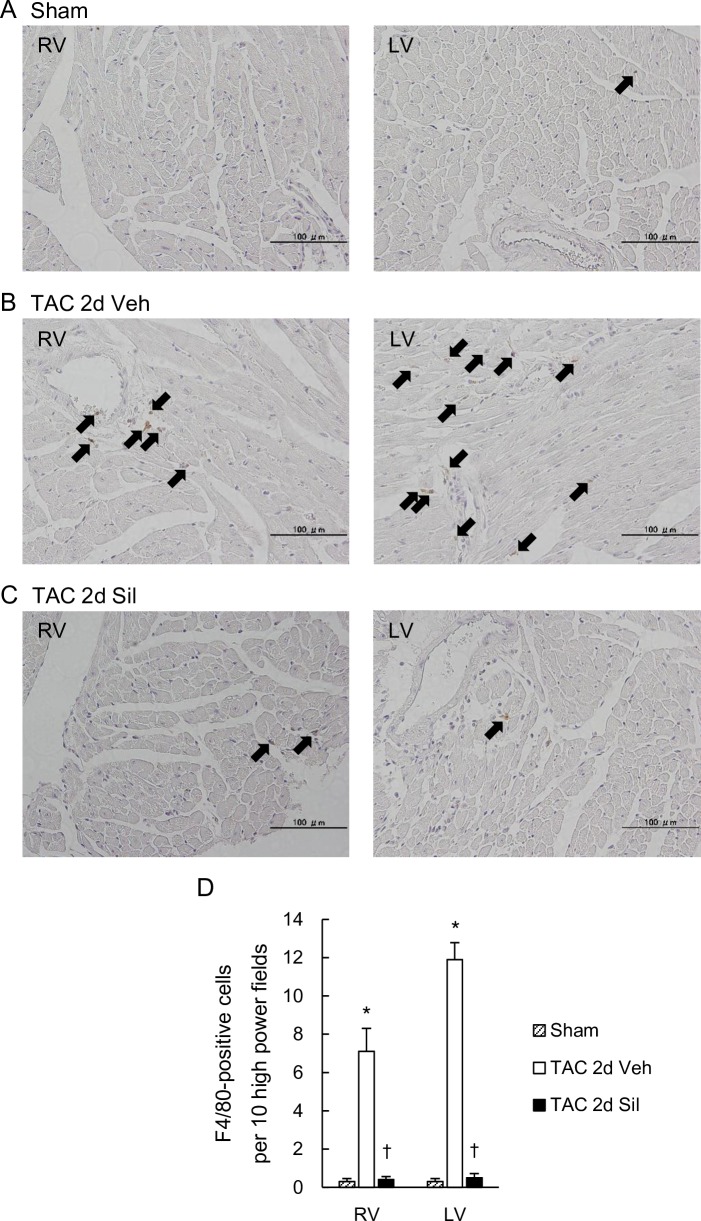
Macrophage infiltration into myocardium was induced also in the RV, which was suppressed by sildenafil. (A-C) Myocardium stained for F4/80^+^ cells in the RV and the LV of the Sham mouse (A), the TAC-2d-Veh mouse (B), and the TAC-2d-Sil mouse (C). Arrows: F4/80^+^ cells. Magnification x200. (D) The number of F4/80^+^ cells per high-power field. Transverse aortic constriction for two days induced F4/80^+^ macrophage infiltration into myocardium not only in the LV but also in the RV, which was suppressed by sildenafil. Results are expressed as mean ± s.e.m. (n = 10). TAC 2d Veh, TAC for 2 days with vehicle treatment; TAC 2d Sil, TAC for 2days with sildenafil treatment. *, p<0.05 versus sham group; ✝, p<0.05 versus the TAC 2d Veh group.

As inflammation marker genes were up-regulated in both ventricles at this early stage, and were prevented by sildenafil, we further performed an immunohistochemical study and assessed macrophage infiltration in the RV and the LV. We found that F4/80 positive cells were significantly increased in both ventricles of 2day-TAC hearts and that sildenafil significantly inhibited the increase in both ventricles (Fig 5).

### Dexamethasone prevented molecular abnormalities in the RV as well as in the LV during LV pressure-overload

**Fig 6:**
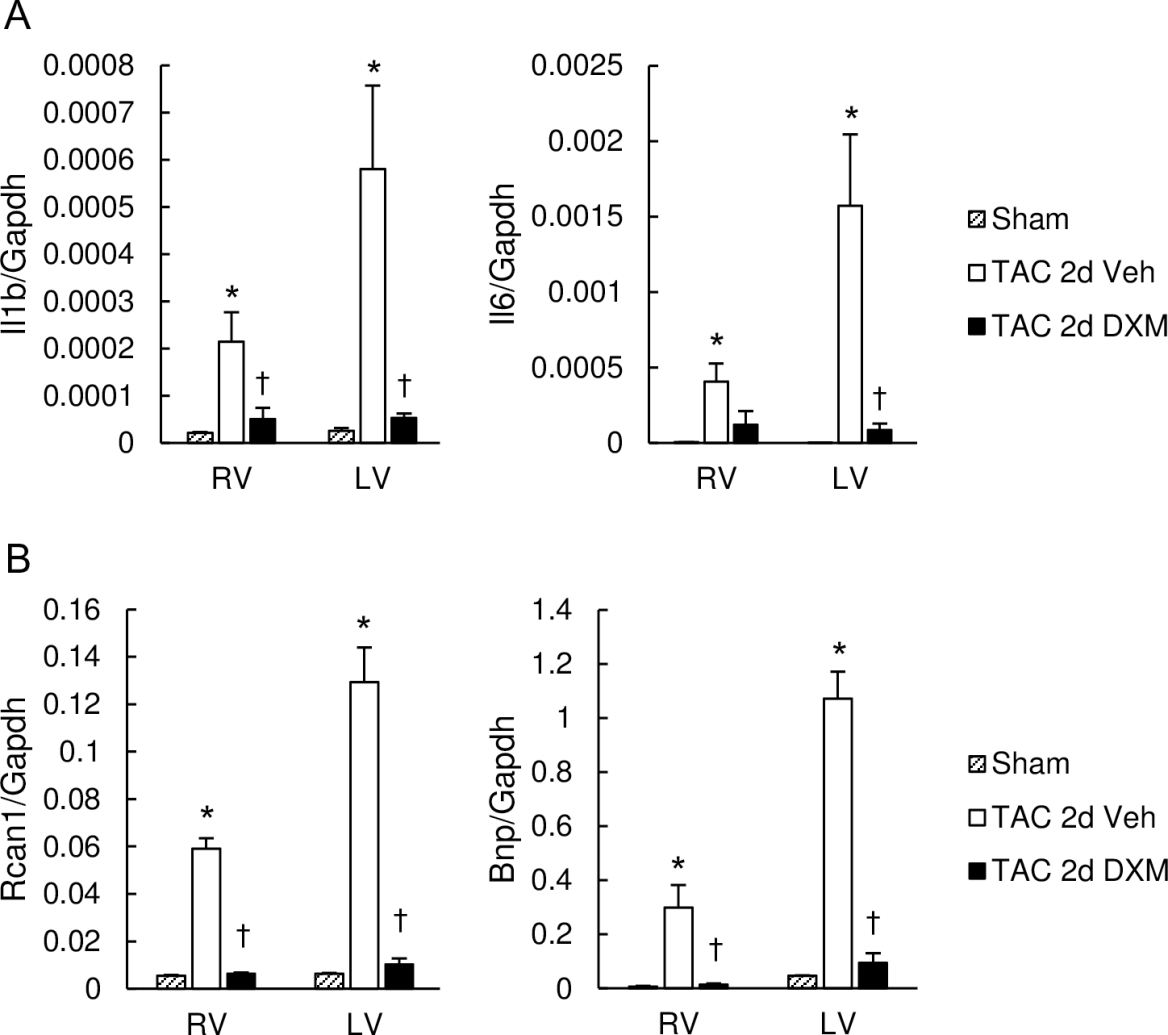
Dexamethasone suppressed pathological molecular remodeling induced in the RV as well as the LV. (A) mRNA expression of IL1b and IL6, normalized to GAPDH. Dexamethasone inhibited overexpression of IL1b in both RV and LV myocardium induced by transverse aortic constriction (TAC) for 2 days. (B) mRNA expression of RCAN1 and BNP normalized to GAPDH. Dexamethasone suppressed up-regulation of RCAN1 and BNP in RV and LV myocardium induced by two-day TAC. Results are expressed as mean ± s.e.m. (n = 5). TAC 2d Veh, TAC for 2 days with vehicle treatment; TAC 2d DXM, TAC for 2 days with dexamethasone treatment. n.s. *, p<0.05 versus sham group; ✝, p<0.05 versus the TAC 2d Veh group.

Studies have documented anti-inflammatory properties of sildenafil [12,22–24], which might be potentially linked to Gq-signal de-activation. We next tested if inflammation might underlie the activation of pathological molecular signaling pathways in the RV during LV pressure-overload. Similar to sildenafil treatment, dexamethasone treatment (20mg/kg/day, intraperitoneally) in two day-TAC hearts inhibited the induction of inflammatory maker genes in the RV as well as in the LV (Fig 6A) and also prevented calcineurin activation and BNP up-regulation(Fig 6B). These results support the potential role for inflammation in this process.

## Discussion

Recent meta-analyses revealed that PDE5 inhibitors improve pulmonary hemodynamics and clinical outcomes in systolic heart failure patients with pulmonary hypertension [25,26]. The present study demonstrates that RV molecular alterations occur very early during LV pressure-overload before RV systolic pressure increases and that these molecular derangement in the RV are inhibited by sildenafil through mechanisms potentially involving anti-inflammation. It is therefore tempting to speculate that earlier intervention with PDE5 inhibitors might confer additional clinical benefits in this pathology.

cGMP-PKG (cGMP-dependent protein kinase) activation by PDE5 inhibition not only induces pulmonary vasodilation but also has direct beneficial impacts on the heart through multiple mechanisms. We and others have demonstrated that cGMP-activated PKG binds to Regulators of G protein Signaling (RGS) 2 and 4 to their activation, deactivating Gq-related signaling in LV myocardium [8]. Koka et al. have demonstrated that PDE5 inhibition with Tadalafil improves mitochondrial energy metabolism and LV cardiac function [11]. More recently, anti-inflammatory properties of sildenafil were reported. Sildenafil treatment is associated with reduced circulating cytokines in patients with diabetes [12] or erectile dysfunction [22]. Sildenafil reduces cardiac and renal inflammation in a mouse model of type I diabetes [23], and in a mouse model of neuro-inflammation [24]. Our data demonstrate such anti-inflammatory properties of sildenafil as potential key contributor to ameliorating early pathological molecular derangement in the RV during LV pressure-overload. The anti-inflammatory effect of sildenafil has been demonstrated in pathological conditions of diabetes [23,27], kidney diseases [28] and neuronal disorders [29]. In particular, intensive studies have been performed in diabetes. Chronic PDE5 inhibition ameliorates diabetic cardiomyopathy and endothelial function in humans [10,30–32], and also shifts adipose tissue cell composition towards a less inflamed profile [27]. In streptozotocin-induced diabetic mice, sildenafil protects endothelial cells and limits cardiac and renal macrophage infiltration [23]. Given that endothelial cells play a crucial role by releasing inflammatory mediators, it is reasonable to speculate that reduction of vascular inflammation by sildenafil might be a significant contributor to ameliorating early molecular derangement in both ventricles in the current study, besides its direct cardiac Gq-inhibitory effects from cGMP-PKG signaling. Importantly, dexamethasone, a corticosteroid, virtually normalized early molecular derangement in both ventricles, supporting the key role for inflammation that could involve cardiac myocytes, vasculature, and adipose tissue. Long-term effects of dexamethasone, however, could be different from those of sildenafil [33], considering the former inhibits both protective or detrimental aspects of inflammation. The anti-inflammatory effects might be coupled to the Gq regulatory action by cGMP-PKG, given that Gq activation is closely linked to inflammation. For example, lack of Gq-inhibitory protein RGS3 in mice reveals exacerbated inflammation in a mouse model of asthma [34]. Also, in a mouse model of chronic kidney disease, RGS2 deficiency results in enhanced fibrogenic and inflammatory response [35]. The precise molecular mechanisms linking Gq signal activation to inflammation and their regulation by cGMP-PKG, however, warrants further investigation.

Signaling pathways for cardiac hypertrophy and failure have been intensively investigated and clarified in LV myocardium or LV cardiac myocytes [17,18]; however, there is limited data available with regard to whether and how similar molecular pathways are at work to contribute to RV pathophysiology [36]. Thus far, most studies have utilized rodent models of pulmonary artery hypertension (PAH) or pulmonary artery banding in order to answer this question and have demonstrated several aspects of distinct RV remodeling in response to afterload stress [37]; however, little has been determined about the RV remodeling process that occur due to LV diseases. Using a short-term LV pressure-overload (TAC) model which presents early stage compensated LV hypertrophy without compromised RV hemodynamics, we found that pathological molecular signaling pathways were activated in the RV free wall myocardium, which might be mediated by inflammation. Interventricular interaction has been reported, but in the opposite pathological settings [38,39]. Sharma et al. reported that LV expression levels of pyruvate dehydrogenase-4 (PDK4) and B-MHC dynamically change during RV hypertrophy development induced by chronic hypoxia [38]. Lourenço et al. observed up-regulation of endothelin-1 mRNA in the LV with impaired LV function also in a rat PAH model induced by monocrotaline [39]. However, it is likely that these LV abnormalities might be directly caused by hypoxia or monocrotaline [40,41]. The present data is the first demonstration that RV-LV interventricular interaction occurs in the absence of direct hemodynamic impacts on the RV, and that this might be mediated by inflammatory process.

We observed that sildenafil inhibited BNP but not B-MHC expression in both ventricles, though both BNP and B-MHC are hallmark fetal genes that are reactivated in pathological hypertrophy and heart failure. This suggests that their activation mechanisms are under different regulations, consistent with the observation by Kong et al.[42]. De-activation of ERK in both ventricles by sildenafil, in particular, might contribute to the former, given that ERK signaling activation has been well-demonstrated to induce BNP expression by acting on the BNP promoter directly or indirectly through increased GATA4 binding activity [43]. B-MHC up-regulation might occur despite deactivation of both ERK and calcineurin given that neither GATA4 (downstream of ERK) nor NFAT3 (downstream of calcineurin) is involved in direct B-MHC gene regulation [44]. B-MHC gene expression is induced by complex effects of transcription factors, including SRF and MEF2 under control of HDACs (histone deacetylases), and PKC-activated TEF1 [44]. In addition, MHC isoform switch is also coordinated by microRNA 208, encoded within an intron of A-MHC gene [44,45]. Our results suggest that sildenafil might not inhibit PKC or signaling pathways coupled to HDAC regulation in both ventricles at this early stage of the LV disease.

In conclusion, we provide the evidence that RV pathological molecular abnormalities associated with LV disease are initiated early even when the LV disease is still at the early stages, and demonstrate that the PDE5 inhibitor sildenafil has potent effects of ameliorating such molecular abnormalities in both ventricles potentially through the anti-inflammatory effects. The study provides a novel insight into our understanding of the RV pathophysiology associated with LV diseases.

## Supporting information

S1 Appendix The data set. (XLSX)

S1 Fig Western blot of p-ERK1/2 in the RV myocardium. (TIF)

S2 Fig Western blot of t-ERK1/2 in the RV myocardium. (TIF)

S3 Fig Western blot of p-ERK1/2 in the LV myocardium. (TIF)

S4 Fig Western blot of t-ERK1/2 in the LV myocardium. (TIF)
