# Supplementary figures and images for: Sildenafil ameliorates right ventricular early molecular derangement during left ventricular pressure overload
Source: PLoS One. 2018 Apr 5;13(4):e0195528. doi: 10.1371/journal.pone.0195528 (PMC5886579; doi:10.1371/journal.pone.0195528)

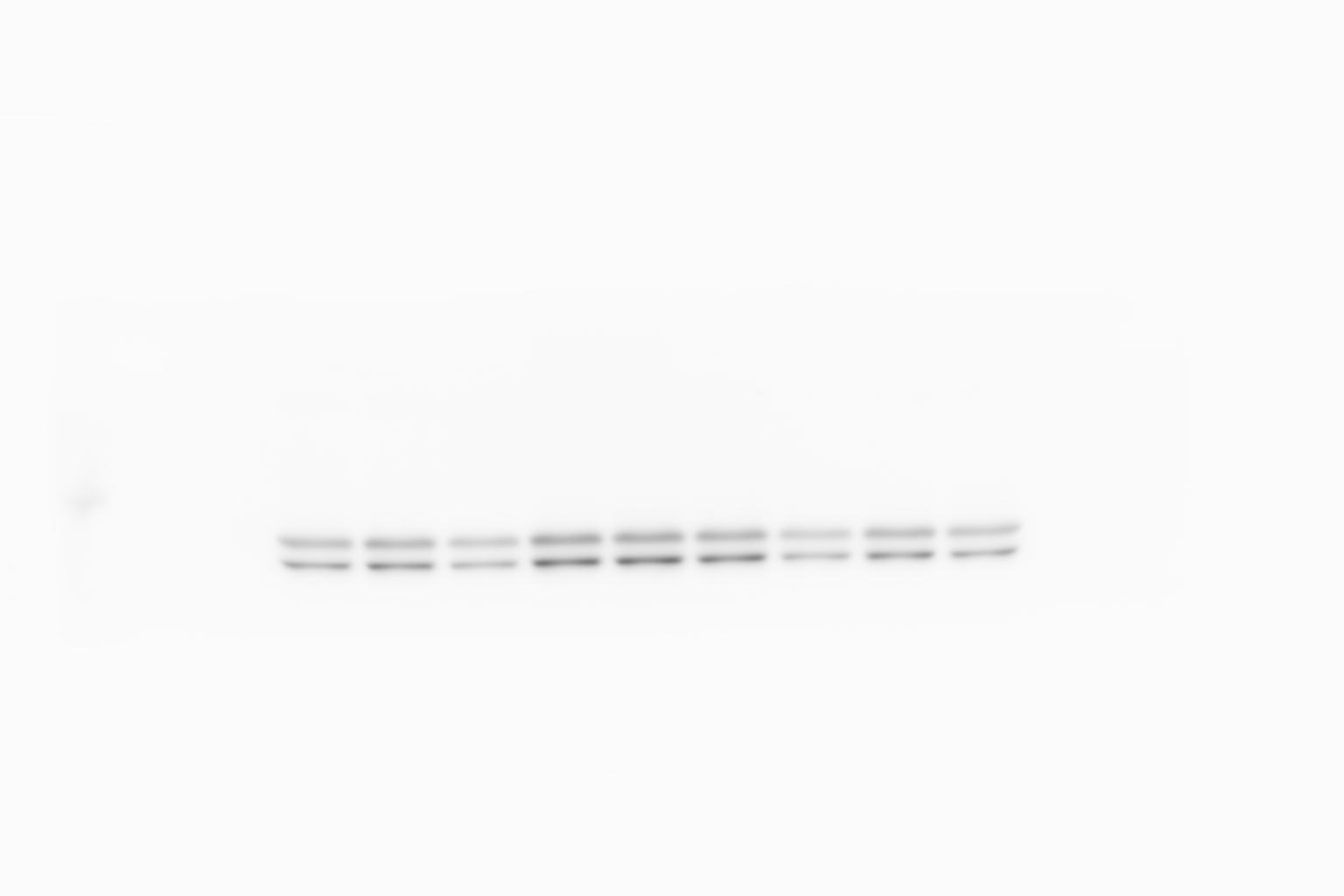

Supplement: S1 Fig — (TIF) [file pone.0195528.s002.tif]

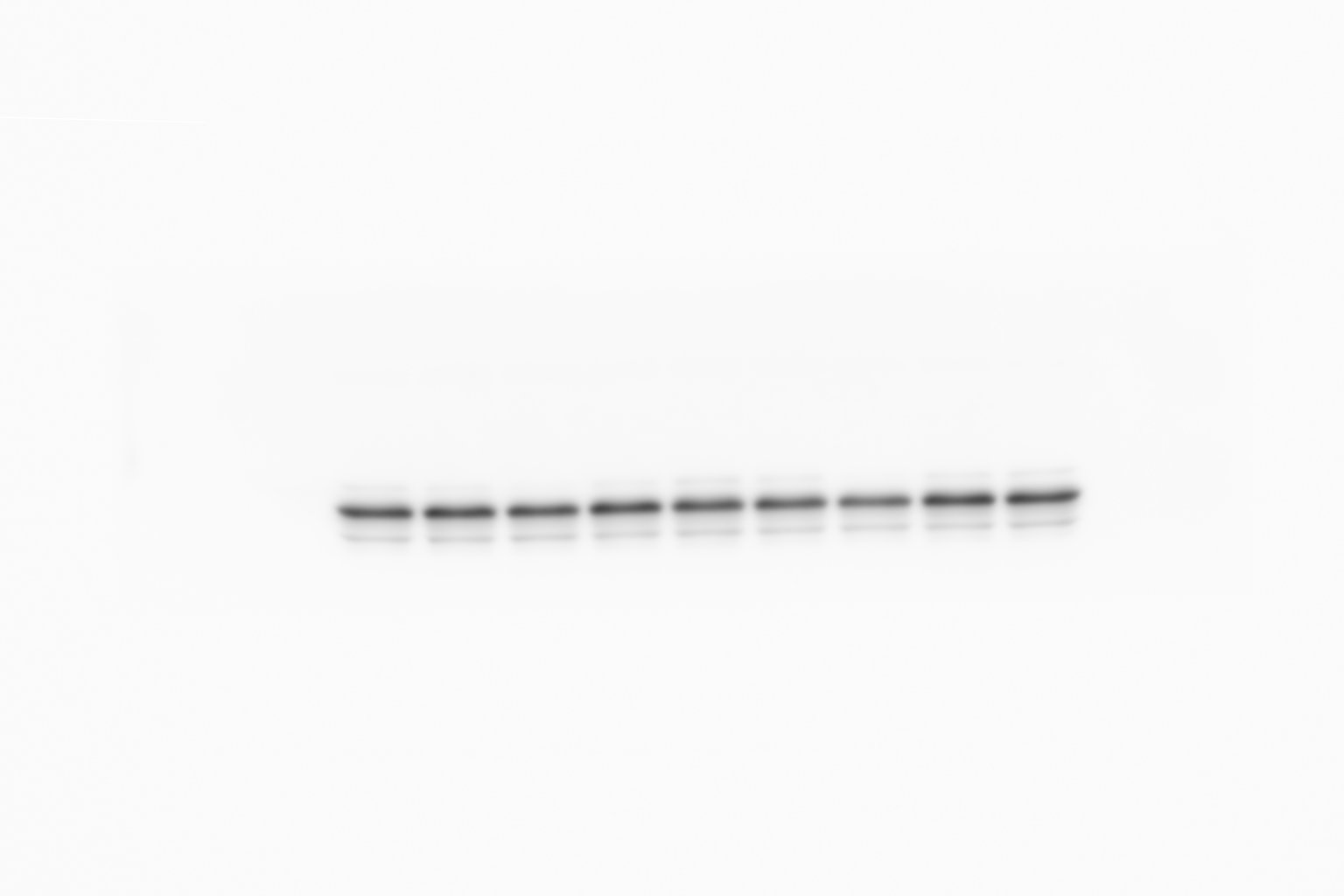

Supplement: S2 Fig — (TIF) [file pone.0195528.s003.tif]

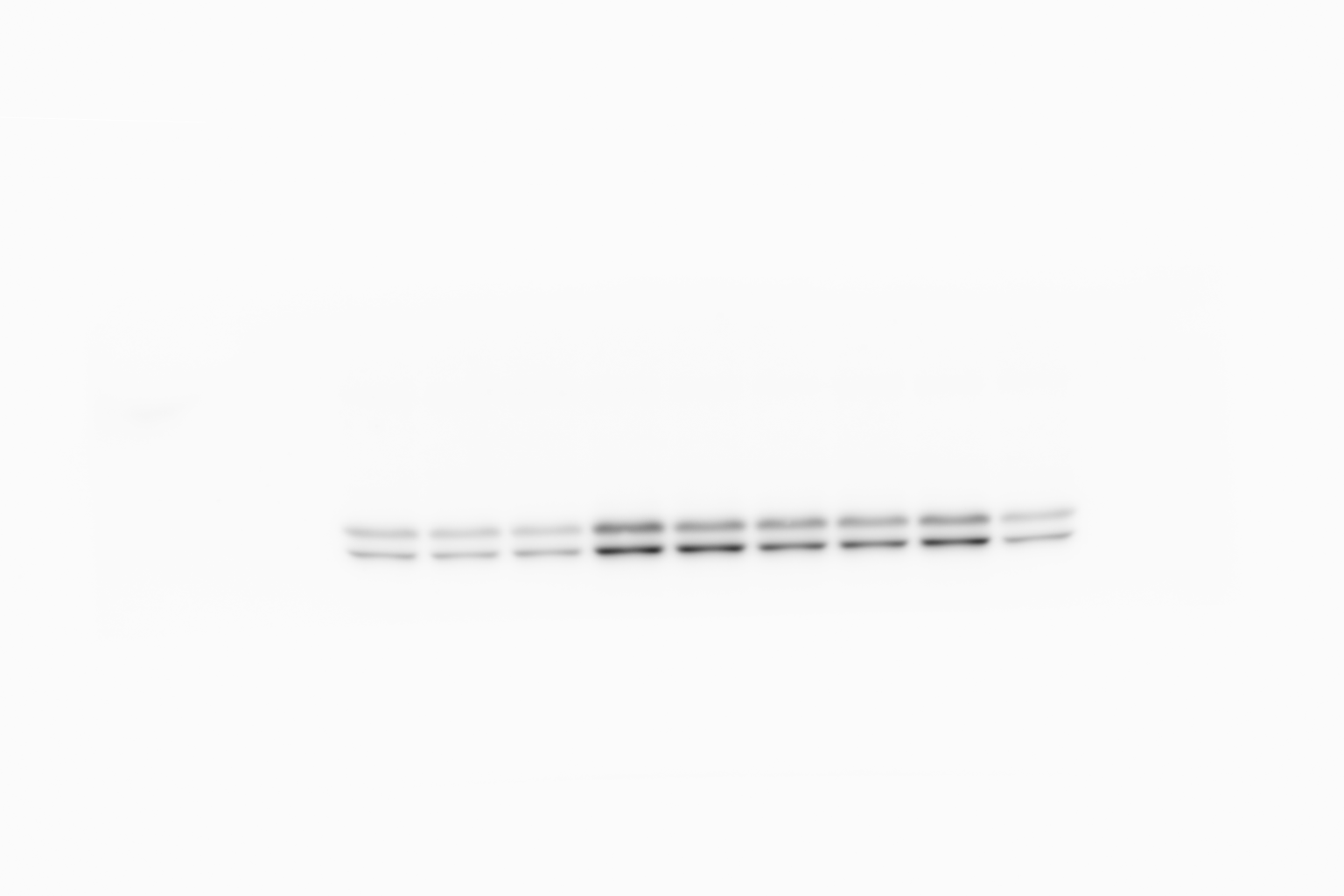

Supplement: S3 Fig — (TIF) [file pone.0195528.s004.tif]

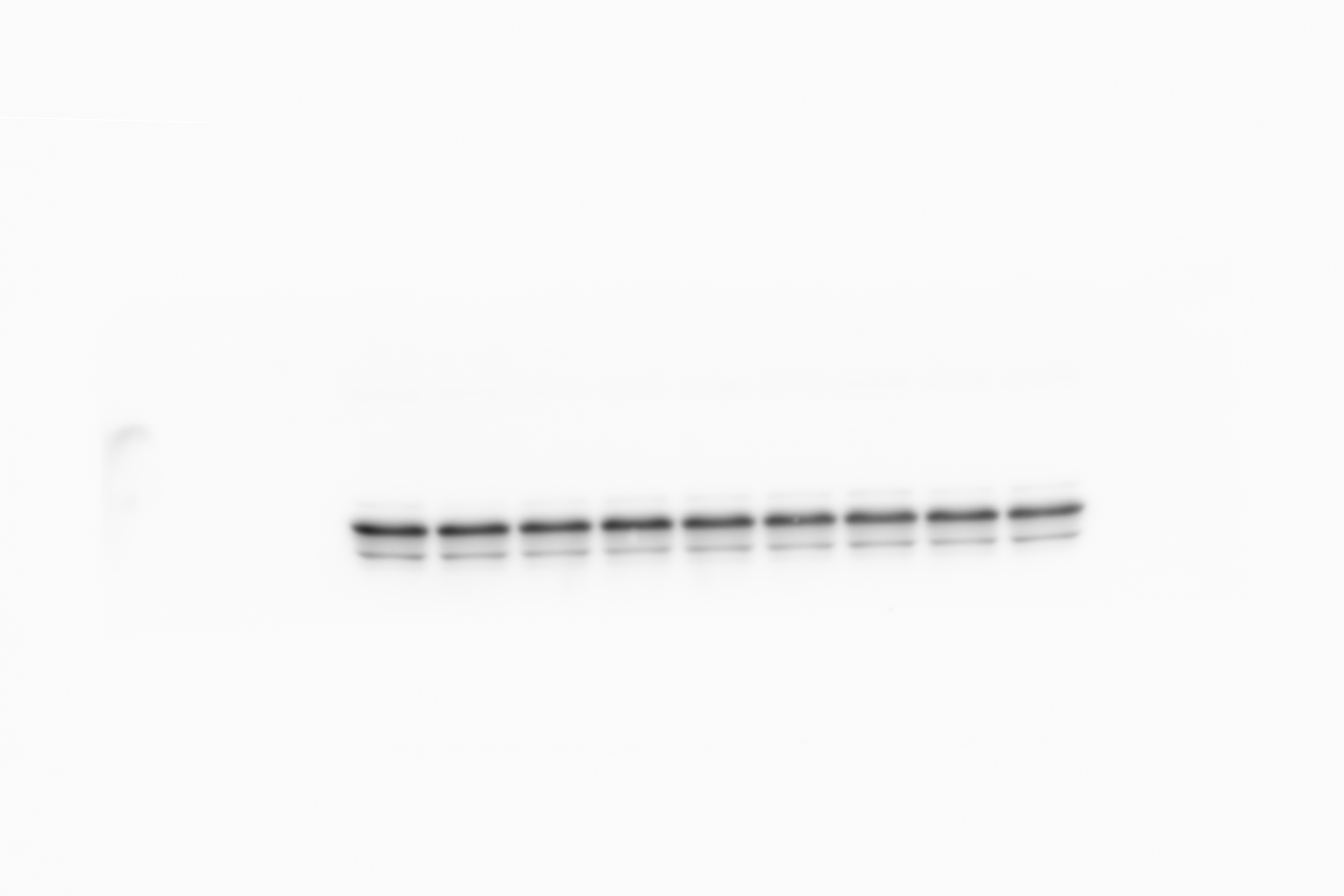

Supplement: S4 Fig — (TIF) [file pone.0195528.s005.tif]
